# On the Role of Artificial Intelligent Technology for Millimetre-Wave and Terahertz Applications

**DOI:** 10.3390/s25175502

**Published:** 2025-09-04

**Authors:** Lida Kouhalvandi, Ladislau Matekovits

**Affiliations:** 1 Department of Electrical and Electronics Engineering, Dogus University, 34775 Istanbul, Türkiye; lida.kouhalvandi@ieee.org; 2 Department of Electronics and Telecommunications, Politecnico di Torino, 10129 Turin, Italy; 3 Istituto di Elettronica e di Ingegneria dell’Informazione e delle Telecomunicazioni, National Research Council of Italy, 10129 Turin, Italy; 4 Department of Measurements and Optical Electronics, Politehnica University Timişoara, 300006 Timişoara, Romania

**Keywords:** artificial intelligence (AI), antenna, millimeter-wave (mm-wave), neural network (NN), optimization, sixth-generation (6G), terahertz (THz)

## Abstract

Next-generation wireless communication networks are developing across the world day by day; this requires high data rate transportation over the systems. Millimeter-wave (mm-wave) spectrum with terahertz (THz) bands is a promising solution for next-generation systems that are able to meet these requirements effectively. For such networks, designing new waveforms, providing high-quality service, reliability, energy efficiency, and many other specifications are taking on important roles in adapting to high-performance communication systems. Recently, artificial intelligence (AI) and machine learning (ML) methods have proved their effectiveness in *predicting*. and *optimizing* nonlinear characteristics of high-dimensional systems with enhanced capability along with rich convergence outcomes. Thus, there is a strong need for the use of these intelligence-based methods to achieve higher bandwidths along with the targeted outcomes in comparison with the traditional designs. In this work, we provide an overview of the recently published works on the utilization of mm-wave and THz frequencies for designing and implementing various designs to carry out the targeted key specifications. Moreover, by considering various newly published works, some open challenges are identified. Hence, we provide our view about these concepts, which will pave the way for readers to get a general overview and ideas around the various mm-wave and THz-based designs with the use of AI methods.

## 1. Introduction

**Figure 1:**
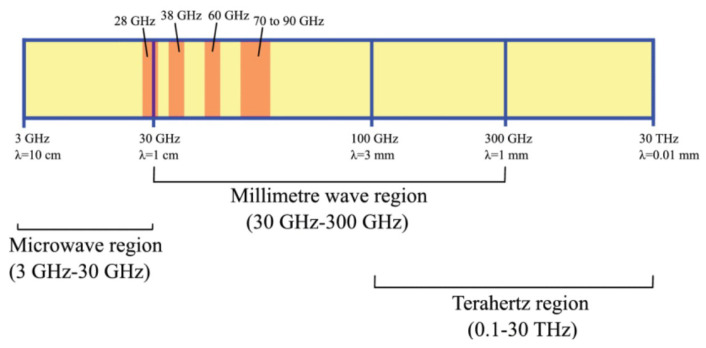
Demonstration of microwave, mm-wave, and THz wave range frequencies presented in [12].

The need for a new wireless communication technology has been raised due to the widespread use of mobile devices and industrial applications [1,2]. The millimeter-wave (mm-wave) and terahertz (THz) frequencies, which are a subset of electromagnetic (EM) waves [3], are identified as the solution for the next generation in high-end applications such as remodeling telecommunications, imaging, and sensing [4,5]. One of the benefits of these high frequencies is enabling localization performance in an accurate and precise way [6] and they can offer high data rates and low latency [7,8]. At various frequency domains, there are diverse applications (Figure 1 represents the various frequency domains). The mm-wave band frequency ranges from 30 GHz to 300 GHz, and the THz band frequency is from 0.1 to 30 THz [9,10]. As is clear from the depicted figure, the THz bandwidth is large enough for transmitting huge data rates and also appropriate for short-range indoor situations [11].

Recently, various studies have been completed in mm-wave and THz frequency bands, especially combined with their effectiveness in various domains such as analyzing large amounts of data, predicting and deciding on channels, self-organization, and so on [13]. It is anticipated to support high data transmission along with high-bandwidth wireless communications, which need advanced methodologies to support advanced wireless applications. To overcome these challenges, the artificial intelligence (AI) method proves its effectiveness in solving problems with big data [14,15,16,17,18,19]. Hence, this methodology is appropriate enough for optimizing high-dimensional and complex designs and networks with resource management [20]. Thus, recently, various studies have been reported in the domain of mm-wave and THz with the help of AI methodologies. In the following, a short list is reported focusing on the used methods by the different authors. For some of these methods, more detailed presentations are provided in the rest of the paper.

In [21], a multiple-input–multiple-output (MIMO) radar system operating at mm-wave is introduced, leading to recognition of a gesture. Here, a convolutional neural network (CNN) based on long short-term memory (LSTM) layers is employed. In another study [22], CNN-based bidirectional long short-term memory (BiLSTM) is employed as a solution for vehicle-to-everything (V2X) communication, leading to an effective beam alignment. For vehicle-to-infrastructure (V2I) scenarios, deep learning (DL) becomes a solution for beam selection in [23]. The reconfigurable intelligent surface (RIS) is one of the successful sixth-generation (6G) technologies for enhancing the overall performance. For this case, in [24], RIS development, along with the utilization of machine learning (ML), is introduced. Federated learning (FL) is also employed in [25] and allows the mm-wave systems to obtain efficient link configuration. As another application of neural network (NN), in [26], this method is employed for predicting the beam signal-to-noise ratio (SNR) from channel state information (CSI) achieved at the base station (BS). In [6], DL-based regression models are executed for predicting range and the angle of arrival (AoA).

In [7], the weighted minimum mean square error manifold optimization algorithm is employed for the MIMO orthogonal frequency division multiplexing (OFDM) transceiver system. This method leads to achieving acceptable spectral efficiency. The mm-wave channel is modeled as a two-state Markov channel in [27], resulting in minimizing the average delay by over 70%. A low-complexity physics-based simulation tool is presented in [28], leading to the training and construction of a network with the mm-wave signal data. In [29], an mm-wave-based unmanned aerial vehicle (UAV) is presented, which operates beyond the fifth generation (5G). Here, a cost-subsidized multiarmed bandit with double-deep Q-network has introduced results in some solutions in terms of the dynamic UAV path design, power splitting, and device pairing. From another point of view for wireless communication systems, path loss modeling is executed through ML in [30]. The implementation of federated learning is presented in [31] for vehicular mobility scenarios, leading to speeding up the sector selection. The graph neural network (GNN) is used in [32] as a beam selection for ultra-dense device-to-device mm-wave networks. Another use of ML is executed in [33] for the purpose of handover reduction in 5G networks.

As presented, there are various types of NNs with diverse utilization targets. Hence, the implementation of AI methods is growing gradually, and it is expected to have technology such as the ’AI internet of everything’ in 2030 [34]. Due to this importance, this work is devoted to firstly summarizing the various reported works based on AI methods that are executed at mm-wave and THz frequencies. Afterward, the authors discuss a future direction regarding this concept. This work will be useful enough for researchers who desire to consider the practical designs, optimizations, and methodologies operating at mm-wave and THz band frequencies.

The remaining part of this work is as follows. The motivation of this work is presented in Section 2. Section 3 and Section 4 present the general definitions of optimization and AI methods, respectively. Section 5 is devoted to summarizing the practical implementation of AI at mm-wave and THz frequencies. Future directions and suggestions of the authors regarding the determined topic are discussed in Section 6. Finally, Section 7 concludes this work.

## 2. Motivation and Scope of This Work

The new radio concept is developing exponentially and attracting the attention of researchers across the world from industry and academia due to its ability to transfer data at a high rate. Hereby, 5G and 6G technologies operating at mm-wave and THz bandwidths have become a hot topic; they are used for various applications. In this case, in recent years, diverse works with comprehensive surveys have been carried out, and Table 1 at the end of this section presents the reported survey works in which the application of AI at higher bandwidths is reported. By reviewing the table, it is recognized that the current presented work, as opposed to the previously published works, focuses on presenting various employed AI-based methods at high frequencies along with enlightening the presented circuit-level/system-level topologies. The benefits of ML techniques lead to improving the overall performance of various designs in diverse applications, as reported as well. Finally, a comprehensive overview of the future directions that can be pursued in this field is presented in detail.

## 3. Definition of Optimization

The optimization method is the collection of mathematical principles leading to solving quantitative problems and finding optimal solutions for various conditions. The optimization process can be executed as either single-objective or multi-objective optimizations as defined in (1):

(1) $$\left\{\begin{array}{c} \mathrm{minimize}\;g\left(x\right);\;g\left(x\right)\in R^{m}\\ \begin{array}{c} \mathrm{subject}\;\mathrm{to}\; \end{array}h\left(x\right)\leq 0;\;h\left(x\right)\in R^{k}\\ x\in \Omega \end{array}\right\}$$

in which:

g(x): Vector with *m* objective functions;
h(x): Vector with *k* constraints;
*x*: Vector with *n* design variables within the design space Ω.

The representation for either a single-objective or multi-objective function can be determined by the *m* factor that, if *m* = 1, the function is single, else the generated function is multi-objective.

**Figure 2:**
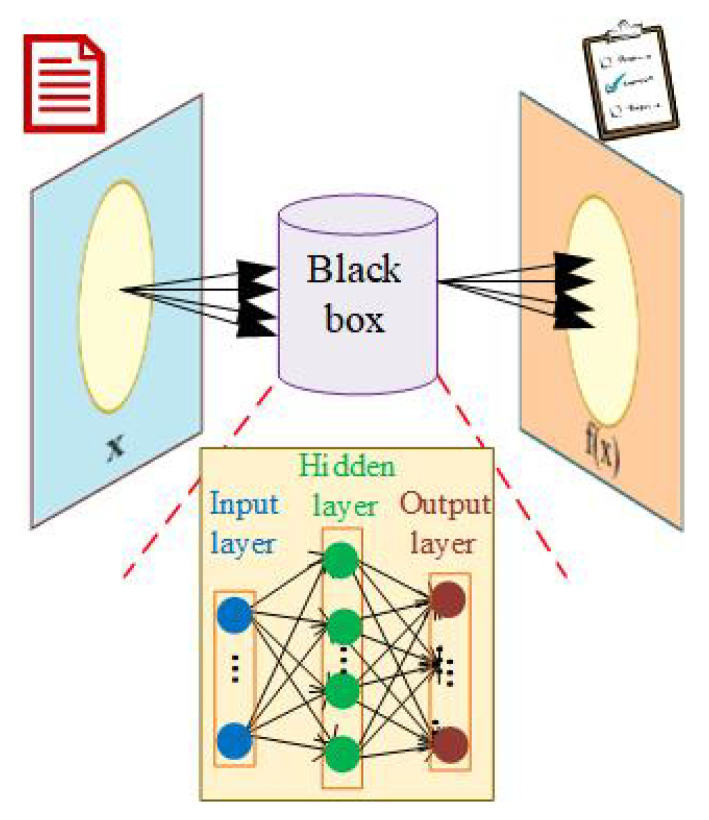
BO method that is employed for ANN training [46].

Bayesian optimization (BO) is a common algorithm used in the AI method and can be defined as either a single-objective or a multi-objective method. By constructing the Gaussian process (GP) regression, any given input data defined as *x* is inserted into the optimization method, and the output parameter defined as f(x) is predicted (see Figure 2). The GP presents a Bayesian posterior probability distribution and aims to estimate the new value by paying attention to the current posterior distribution.

For training any kind of NN, firstly, a suitable amount of data is prepared, and after that, the GP model is generated using the maximum a posteriori (MAP) metric as (2),

(2) $$\omega_{MAP}=argmax\prod_{i=1}^{n}\rho \left(y_{i}|f\left(x_{i};\omega \right)\right)\rho \left(\omega \right)$$

where ρ(ω) represents the prior probability with the weighting vector: $\omega =(\omega_{1},\omega_{2},\ldots ,\omega_{m})$. Also, *x* and *y* parameters are defined as input data and predicted output response, respectively. After obtaining the required MAP, the $x_{t}=argmax\left[EI\left(x\right)PI\left(x\right)\right]$ is chosen, where EI is the expected improvement and PI is the probability of improvement.

**Figure 3:**
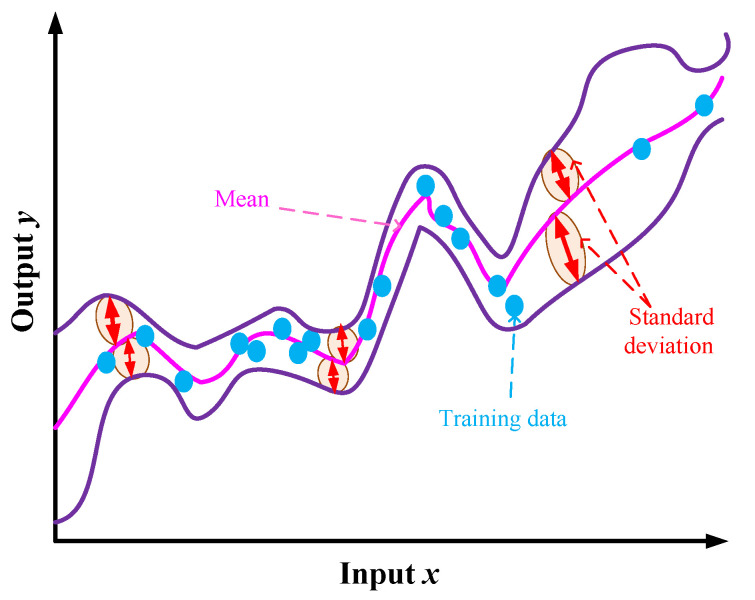
The representation of GP model leading to predicting the new outputs for the given input data.

For the newly inserted input, the new output is estimated through the Gaussian distribution, as presented in (3) and (4), with a kernel function of $K(x,x^{\prime })$, a mean m(x), and a standard deviation σ(x). In case of not achieving the targeted output, the GP model is updated to achieve the required specifications. Figure 3 presents the performance of GP for estimating the new outputs.

(3) $$m\left(x_{n+1}\right)=K^{T}{\left[K+\sigma_{n}^{2}I\right]}^{-1}Y$$

(4) $$\sigma^{2}\left(x_{n+1}\right)=K\left(x_{n+1},x_{n+1}\right)-K^{T}{\left[K+\sigma_{n}^{2}I\right]}^{-1}K$$

## 4. Definition of Artificial Intelligence

**Figure 4:**
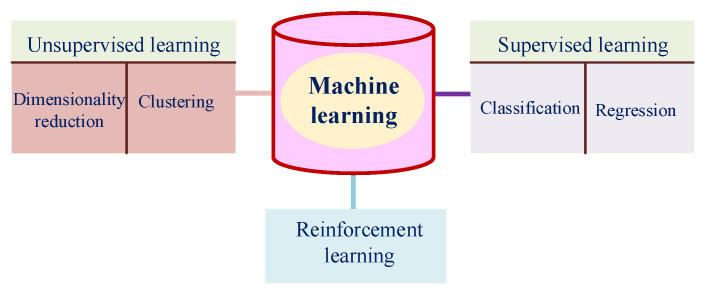
Subsections of the ML.

The AI method aims to mimic human intelligence, which involves ML, NN, and DL subsections. For this method, the BO algorithm is employed to estimate the output specifications for any given input data by reducing the error function (i.e., loss function). As Figure 4 shows, the ML is divided into three subsections: supervised learning, unsupervised learning, and reinforcement learning (RL). More descriptions regarding each of the learning methods are presented in [47,48].

**Figure 5:**
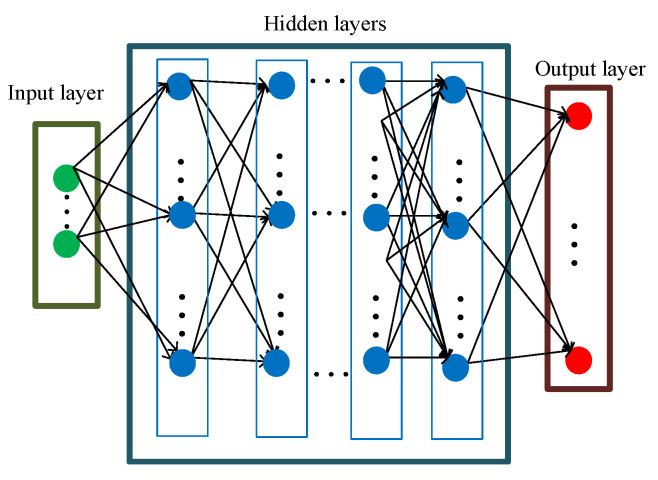
General structure description for DNN.

The structure of any NN can be either a shallow neural network (SNN) or a deep neural network (DNN), including the input layer, hidden layers(s), and output layer. The number of hidden layers will cause the construction of either the SNN or DNN structures. Figure 5 presents the general configuration of DNN, including the input layer, hidden layers, and output layer. Any network can be trained by firstly achieving a suitable amount of data (i.e., training, validation, and testing data), and then by determining the specification(s)/parameter(s) for input and output layers, respectively. The training procedure for networks is described more in [49]. The intelligence-based networks are able to both handle uncertainty and analyze a large amount of data for estimating outcomes [50]. For any reported problems, selecting the appropriate specifications for the input layer, output layer, and the topology of hidden layers can result in accurate predictions and optimizations.

## 5. Application of AI at mm-Wave and THz Band Frequencies

The AI methods are applicable for analyzing and estimating communication data for various systems and wireless environments. Some of the various AI-based algorithms that can be employed for the systems at mm-wave and THz bandwidths are supervised learning, unsupervised learning, DL, and RL [51]. For this case, this section is devoted to summarizing the various methodologies employed for the various designs at mm-wave and THz frequencies. Table 2 at the end of this section summarizes various types of NNs used in high frequencies.

### 5.1. Application of AI at mm-Wave

In modern wireless communications, wireless network planning, along with optimizations, is substantial for finding the optimal network deployment. High frequencies, such as the mm-wave frequency bands, are subject to reflections and diffraction effects. The mm-wave frequencies result in increasing bandwidth with the need for highly directional antennas [52]. Recently, ML algorithms have been employed that offer significant advantages for various concerns [53]. This section is devoted to presenting the employed diverse AI-based algorithms at mm-wave frequencies for designing and optimizing networks.

**Figure 6:**
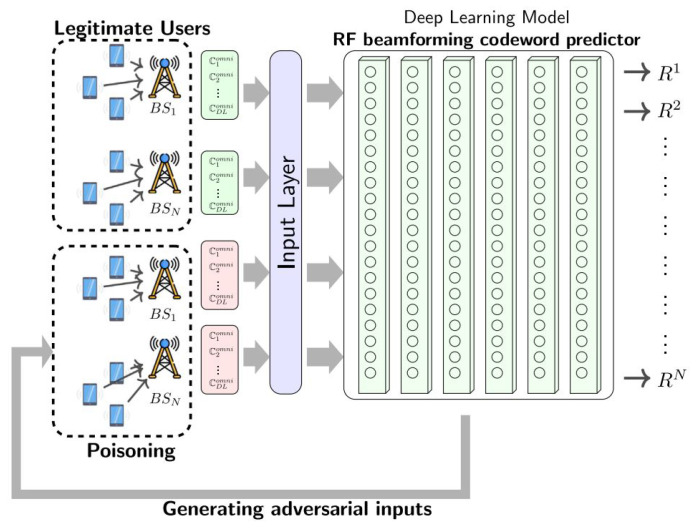
A configuration for beamforming codeword adversarial training presented in [54].

In [54], a methodology for adversarial attacks against the proposed 6G ML is presented that helps in mm-wave beam prediction. Figure 6 presents the diagram for the adversarial training process that is based on DL modeling. Adversarial inputs are generated after the model is constructed, and the model becomes stable when the training is finalized. After this process, the trained model will estimate the radio frequency (RF) beamforming codeword for appropriate users.

In another study [55], the ML method is combined with the genetic algorithm (GA) for finding the network deployment, suitable for 5G wireless systems. The presented method leads to providing at least 95% of the receiver (Rx) point that will be useful enough in the combination of massive MIMO and beam-forming for exposure-aware network planning. For 6G mobile networks [56], the internet of vehicles is an important target, also dealing with green communication. For this case, in [57], the ML method is employed, aiming to improve the energy-efficient concept. Here, the mm-wave channel is modeled by using the 3D ray tracing, which results in reflecting the RF-domain digital twin matching. The presented method, based on the ML method, leads to present connections between the twin network and physical network and helps to improve efficiency and consistency. The ML method is employed in [58] for accelerating the spatial-domain beam prediction, allowing for minimizing the power consumption and the reference signaling overhead. The presented NN includes input specifications as reference signal received powers (RSRPs) and output specifications as $\hat{P}$ that are the predicted output probability vector of all the beams. The presented network is fully connected, including *F* nodes.

**Figure 7:**
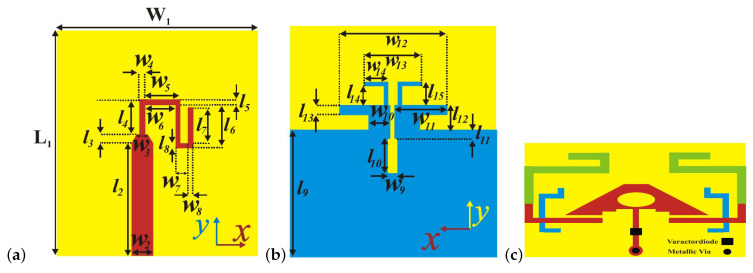
Presented (**a**) front side and (**b**) back side of antenna, (**c**) filter configurations presented in [60].

For calibrating mm-wave on-chip circuits, a methodology, namely a thru-reflect-line NN, is employed in [59], allowing more accuracy and flexibility in comparison with conventional thru-reflect-line methods. This ML-based method estimates the S-parameters of the device under test. This method is validated for calibrating the transmission lines for the whole D-band of 110–170 GHz. In [60], a 5G mm-wave dipole antenna is designed and fabricated, allowing a high gain of 6.7 dBi at 28 GHz. Additionally, a 5G filter with a gain insertion loss lower than 0.6 dB is presented. For the designed phased-array structure, ML with the collaboration of BO is executed in the hybrid beamforming, leading to achieving suitable gains in analog and hybrid beamforming. The general configuration of the antenna with filter is presented in Figure 7.

The DL method is presented as a solution in [61] for achieving the channel state information of mmWave massive MIMO systems. The presented method leads to selecting the appropriate beam from the base station to the user. The type of employed NN is long short-term memory, which allows for selecting the suitable beam. For detecting the mm-wave channel states and scenarios, in [62], a DNN-based approach is presented in which the identification is improved effectively by using the cluster-based features. The effectiveness of the method is verified in both line-of-sight (LoS) and non-LoS (NLoS), which are channel states.

**Figure 8:**
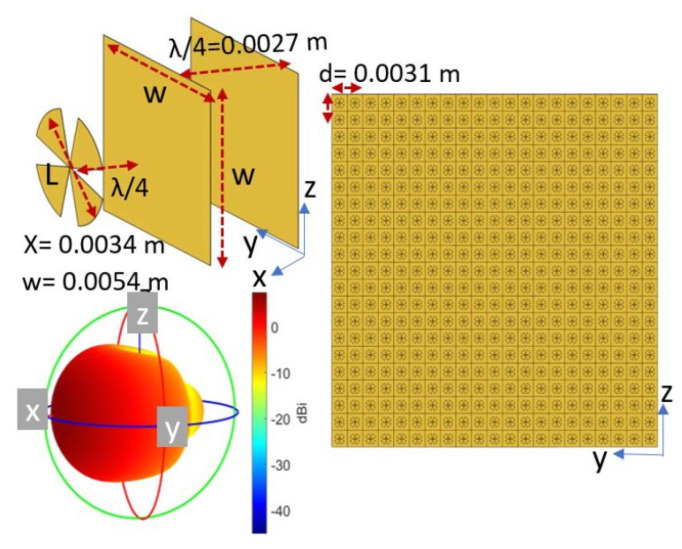
MIMO antenna configuration presented in [64].

Achieving training data for modeling the mm-wave channel predictors with NN, and also generating an accurate model, faces limitations. For tackling these drawbacks, in [63] an automatic hyperparameter tuning method is employed, which leads to optimizing the training process of the presented CNN method. The ML-based beamforming method is employed in [64], which is executed with the k-nearest neighbors approximation. The approach allows for generating suitable beamforming configurations with respect to the spatial distribution. With this method, a direct mapping is performed, and the beamforming complexity is minimized effectively. The general structure of the presented antenna array is presented in Figure 8.

The supervised learning method is employed in [69] to execute beam sweeping in a low implementation and to perform a binary classification. Here, model training is performed by extracting the features from raw data. Unsupervised learning is executed in [65], leading to the estimation of the beamspace channel for mm-wave MIMOs. This methodology improves the compressive sensing technique successfully.

In RF-based user authentication, there is an important problem of behavior privacy leakage. For this case, a DNN, namely BPCloak, is presented in [66], leading to the erasing of behavioral privacy. This technique has been verified in mm-wave systems. In another study [67], reconfigurable intelligent meta-surfaces (RIMs) are designed to improve the beam steering functionality suitable for 5G communications. Here, the computed vision (CV) system runs the ML method to calculate in real time the coding schemes, which results in achieving the targeted reconfigurable radiation pattern. Additionally, the effectiveness of the GA in achieving the most appropriate radiation pattern for beam steering application is presented. In [68], a new method for sideline-assisted multiple-mode mm-wave scheduling is presented. Forming the multicast clusters would help in reducing the complexity, and the unsupervised learning algorithm helps improve transmit power and bandwidth specifications.

**Figure 9:**
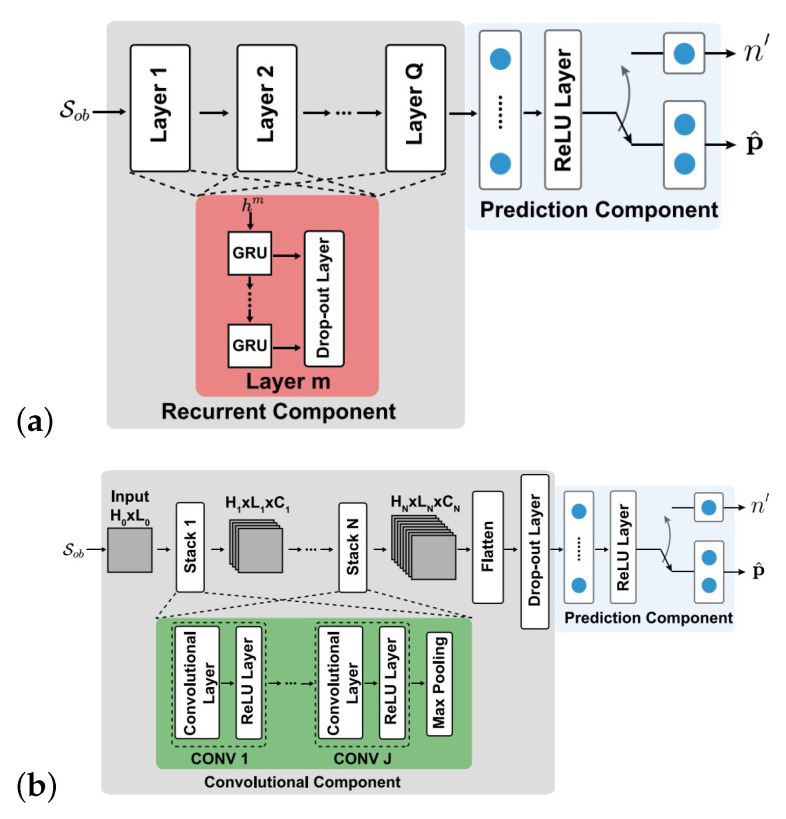
(**a**) RNN and (**b**) CNN structures presented in [70].

For estimating the future dynamic line of sight link blockages, in [70], DNN is employed, which results in 85% accuracy. In this study, the link blockage status is estimated by the recurrent neural network (RNN), and also the link status with the type and moving direction of the blockage through CNN. The structure of RNN and CNN is depicted in Figure 9, respectively.

Based on the mm-wave radar and ML, in [71], a noncontact carry object detection methodology is presented. Here, a tree-based feature selection method is executed to minimize the complexity and enhance reliability. In another study [72], the DL method is employed, resulting in presenting a solution for precise 5G positioning. The problem is modeled as the combination of non-line-of-sight identification and position estimation. The Markov decision process problem is solved in [73] by learning from the environment along with re-optimizing the caching policy. Here, the RL is employed and trained to generate a change point detection-assisted reinforcement learning algorithm. It is verified that the presented method has a higher convergence speed in comparison with a conventional Q-learning algorithm.

A link adaptation framework for the orthogonal frequency-division multiplexing systems with compressed-sensing-assisted index modulation is presented in [74], in which the methodology is based on the DL. In this work, multi-layer sparse Bayesian learning algorithms with DNN-assisted adaptive modulation algorithms are executed, leading to the accurate optimization of channel knowledge and transmission mode. The mm-wave MIMO system is presented in [75], in which the presented method is based on the DL results in hybrid precoding. The constructed network is CNN, which estimates and optimizes the hybrid precoder and combiner. In another study [76], a new receiver structure, implementing DL at the receiver side, presented results in detecting mm-wave radio-over-fiber signals. This framework outperforms the bit error rate from $10^{-1}$ to $10^{-5}$ in comparison with the conventional self-homodyning-based approach. The beam training concept is studied in [77] for the MIMO systems. In this work, the DNN is employed for estimating the strongest channel path based on the probability vector, and also, this NN is used for executing beam training tests. The outcomes demonstrate that enhanced signal coverage can be achieved.

**Figure 10:**
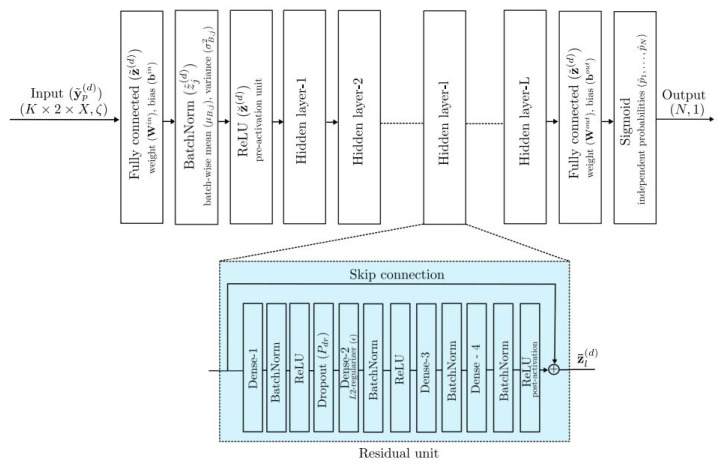
A general structure of neural network presented in [78].

**Figure 11:**
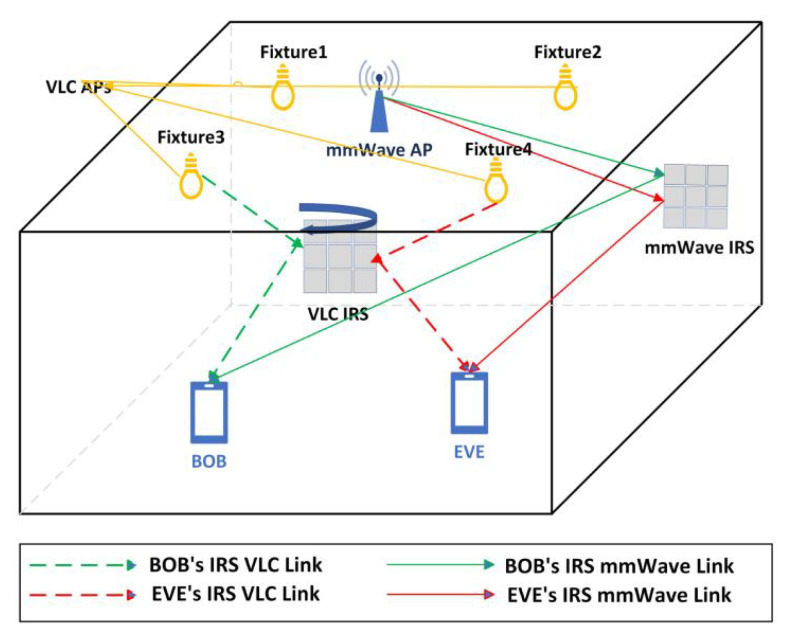
A general view of mm-wave system, modeled by intelligent reflecting surfaces (IRS)-assisted visible light communication (VLC) presented in [80].

In [78], a pre-activated residual NN-based multi-user detection is presented (see Figure 10). The presented network leads to solving the sparse signal recovery problem in massive machine-type communications. The beam prediction NN is presented in [79] for beam management in non-cooperative mm-wave systems. The novelty of this work relies on the execution of fewer training samples in comparison with the conventional methods, in which deep regularized waveform learning is employed. In [80], a deep reinforcement learning method is performed based on the deep deterministic policy gradient methodology. This method is able to comply with channel variations, resulting in improved secrecy capacity of the authorized receiver. Figure 11 presents communication channels with the presented model, leading to outperforming the overall performances, in which the intended recipient refers to BOB and the eavesdropper refers to EVE.

**Figure 12:**
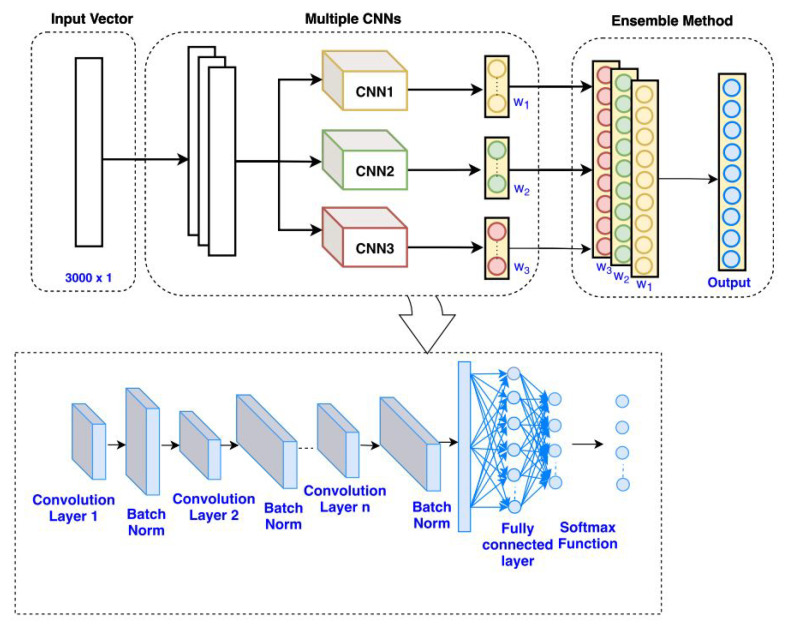
CNN-based methodology presented in [81].

In [81], a CNN classifier method is employed for solving problems such as sensing and localization challenges in indoor scenarios. The general configuration of NN is presented in Figure 12, which includes three CNNs, and batch normalization is executed for all of the convolutional layers. The presented network results in training accuracy of 97.65% and testing accuracy of 96.47%.

**Figure 13:**
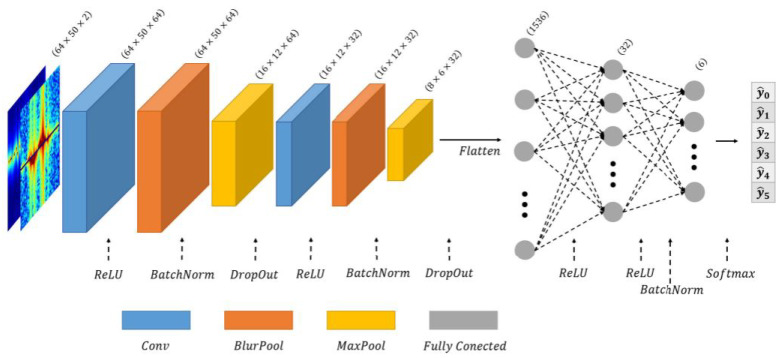
Gesture classification with CNN employed in [82].

The mm-wave radar sensors are also used for gesture recognition. In [82], finger-level gesture recognition with the help of mm-wave radar is presented. In this study, the ML technique is employed, and with this method, the activity detection module and gesture classifier are constructed, resulting in an accuracy of 95%. The detailed description for the configuration of executed CNN is presented in Figure 13.

As previously presented, the RIS system is generally used for mm-wave positioning systems. In [83], a residual convolution network regression is employed for predicting the three-dimensional locations of the mobile users in MIMO systems. The simulation results show an acceptable training accuracy of more than 90%. With the help of mm-wave radar, dynamic gesture recognition is executed in [84], leading to reduced model complexity. Based on the mm-wave radar, a posture classification system through the AI solution is introduced in [85], which uses two types of data: an image dataset along with a spatial coordinate dataset. ϵ-Fuzzy Pareto active learning is employed in [86] for mm-wave MIMO systems, resulting in low complexity with low iteration convergence. Here, hybrid precoding, fully connected with partially connected structures, is taken into account. In another study [87], instead of implementing DNN results in limited training data along with real-time models, broad learning is executed for the problems of beam alignment used for mm-wave cell-free MIMO downlink systems. Mobility management is also another important issue for which, in [88], computer vision is employed for estimating the blockages, leading to the timely performance of the handover. With this method, a 40% improvement is achieved in comparison with the conventional ones. For the mm-wave mobile system, the benefit of GNN is presented in [89], which leads to learning end-to-end hybrid precoding through a parallel proactive optimization network. This type of network includes several graph neural networks that can be generalized across diverse system configurations. In [90], the ML method is executed for estimating the future beams through light detection and ranging (LiDAR) sensors. This LiDAR-aided beam prediction is able to estimate the optimal beam with 95% accuracy.

In [91], a long short-term memory (LSTM)-based method is executed for estimating the best-performing beam in terms of magnitude for both analog and digital beamforming structures. Due to the recent importance of beam codebooks [92], in [93], NN is trained for translating a codebook into a consistent beamspace. An ML-based cross-entropy (CE) method is employed in [94] for optimizing active and passive beamforming. For network planning with 5G, a highly accurate pass loss estimation is required, and in [95], the ML-based urban canyon is presented. The mm-wave indoor multi-user communications through an ML-based vision-aided beam selection are presented in [96], leading to the selection of an optimal beam.

### 5.2. Application of AI at THz

The prospective 6G communications need a large bandwidth with massive data rate support. The use of AI and ML methods at THz band frequencies has been reported in various works in recent years, leading to tackling problems in aspects such as channel estimation, beam control, spectrum management, and so on. This section is devoted to presenting the very recently published studies on this issue.

**Figure 14:**
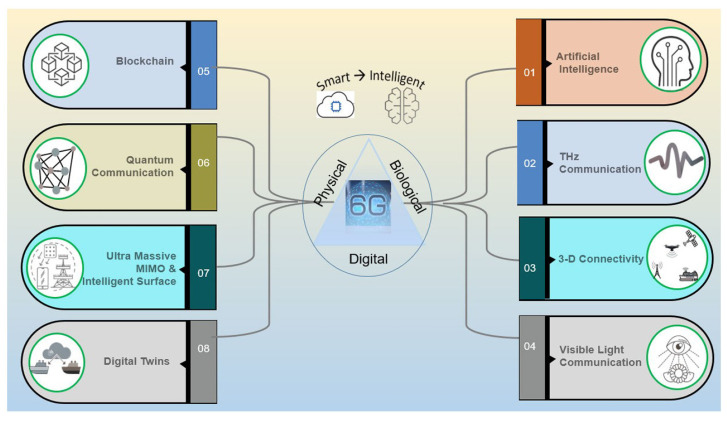
Diverse domains of applicability of 6G technology presented in [97].

In the telecommunication industry, there are various studies on the development of 6G, especially from the view of the 6G ecosystem [97]. This 6G technology can be executed in various domains, as depicted in Figure 14. The utilization of the THz frequency band for 6G communication has become popular recently due to its high data rate transportation, which is also important for AI science. This new frequency band could be developed for quantum communications as well as predicted in [98]. In [99], the intelligent reflecting surface (IRS) is presented, which operates in the THz frequency band and is able to generate a controllable propagation environment. In this work, the DL-based channel estimation method is introduced by providing a solution for the sparse recovery problem. In another study [100], a framework based on AI is presented, leading to operation at THz bandwidth. This methodology helps in improving base stations and LoS communication. The simulation results demonstrate the 61% improved results in comparison with conventional channel state information. With the help of the DL method, in [101], a methodology for following the IRS reflection coefficients is presented that is useful for THz communication systems. Here, a liquid state machine with an ensemble learning technique for achieving the targeted specification is employed. The THz signal identification is studied in [102], which results in specifying the glass fiber-reinforced polymer, which solves problems in THz nondestructive testing. In this work, various THz signals are classified.

**Figure 15:**
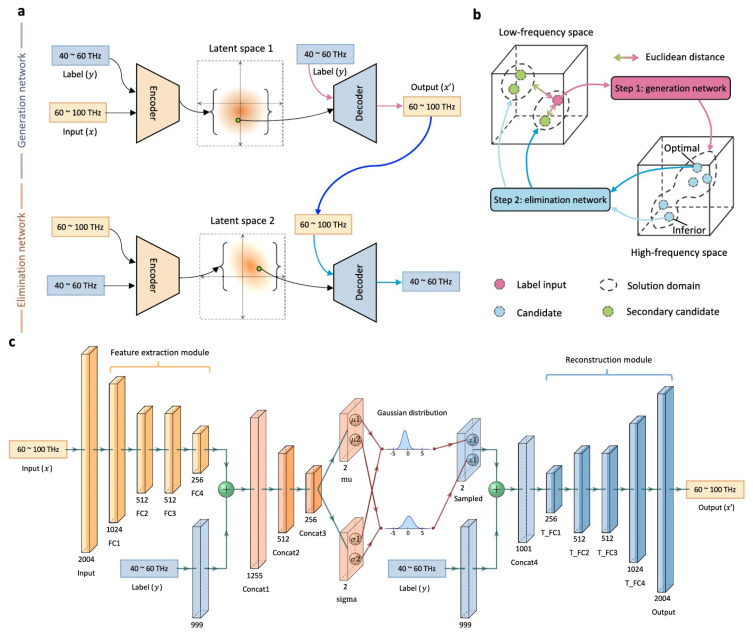
DL-based network architecture presented in [103]: (**a**) general network architecture, (**b**) a process of bidirectional mapping between low-frequency and high-frequency spaces, (**c**) a detailed description of various constructed layers.

The THz metasurface structures are presented in [103] to link the reflection spectra from low to high frequencies. For this case, a DL method is employed with which inaccessible spectra are predicted with an accuracy of 98.77%. The general configuration of the presented network is depicted in Figure 15. The NN structure involves a generation network and an elimination network, where the first network is used as a generator to manufacture various candidates and the second network is used as an inspector to select the optimum solution.

## 6. Future Directions About This Concept

As previously introduced, the mm-wave and THz band frequencies are essential for 5G and next-generation networks. These technologies need large antenna arrays at both the transmitter and receiver sides. For this large number of antennas, achieving optimal beams is one of the important challenges. From another point of view, data rates, latency, energy consumption, reliability with security, and scalability are other issues that must be considered in these bandwidth ranges. Based on the summarized works in this manuscript, some of the nominated drawbacks are considered, and solutions are presented. However, what is missing is the use of intelligence-based networks of various types, along with the implementation of multi-objective optimization methods. Implementation of optimization methods can help in achieving the optimal parameters, resulting in the best matching targets [104].

From another point of view, the AI-based network must be trained by paying attention to the hardware constraints, for which a suitable execution environment in terms of power and memory must be provided. Additionally, the appropriate set of data in terms of quality, privacy, and ethical considerations must be satisfied in order to train an accurate network.

### 6.1. Multi-Objective Optimization

Optimization methods are employed for minimizing and/or maximizing targeted functions. By reviewing recent publications, it can be observed that system-level and circuit-level designs are benefiting greatly from multi-modal optimizations, leading to compact, low-cost, and high-performance structures and specifications. Hence, optimizations can be useful in terms of speeding up the process, improving targeted performances, minimizing errors by employing programming constructs, and arranging collaboration of device-to-device communications. The various optimization methods can also be employed at the output layer section for optimizing the various executed output specifications.

The optimization methods can be divided into three subsections:

1. Algorithms based on animals, plants or insect behaviors (bio-inspired) include:
  - Particle swarm optimization (PSO) [105];
  - Ant colony optimization (ACO) [106];
  - Artificial bee colony (ABC) [107];
  - Artificial fish swarm algorithm (AFSA) [108];
  - Artificial plant optimization algorithm (APOA) [109];
  - Chicken swarm optimization algorithm (CSO) [110];
  - Bacterial foraging optimization (BFO) [111];
  - Firefly algorithm (FA) [112];
  - Fruit fly optimization algorithm (FOA) [113];
  - Wolf pack algorithm (WPA) [114];
  - Shuffled frog leaping algorithm (SFLA) [115];
  - Cuckoo search algorithm (CSA) [116];
  - Bat algorithm (BA) [117].
2. Algorithms based on human treatments:
  - Harmony search algorithm (HS) [118];
  - Social emotion optimization algorithm (SEOA) [119];
  - Teaching-learning-based optimization (TLBO) [120].
3. Algorithms based on evolution processes:
  - Genetic algorithm (GA) [121];
  - Gene expression programming (GEP) [122];
  - Evolutionary programming algorithm (EPA) [123];
  - Genetic programming algorithm (GPA) [124];
  - Multi-objective evolutionary algorithm (MOEA) [125];
  - Memetic algorithm (MA) [126];
  - Differential evolution algorithm (DEA) [127].

### 6.2. Type of NNs

An evolutionary algorithm is an evolutionary AI-based computer application that aims to predict future responses after accurate training [128]. There are various types of networks, as listed below; hence, with respect to the applicability of each one, they can be employed for targeted specifications.

- Recurrent neural network (RNN) [129];
- Long short-term memory (LSTM) [130];
- Gated recurrent unit (GRU) [131];
- Auto encoder (AE) [132];
- Denoising AE [133];
- Markov chain (MC) [134];
- Deep convolutional network (DCN) [135];
- Generative adversarial network (GAN) [136];
- Deep residual convolutional network (DRCN) [137];
- Support vector machine (SVM) [138];
- Recurrent neural network (RNN) [139];
- Transfer learning neural network (TLNN) [140];
- Reinforcement learning (RL) [141].

### 6.3. Implementation of Quantum Computing (QC)

Quantum computing (QC) is able to solve complex optimization algorithms in which a large amount of data is processed. As previously disclosed, 6G communication systems require suitable and sufficient data; hence, the QC can be an advanced solution for large band frequency [142]. From another point of view, the implementation of QC will lead to speeding up the training process of ANN, as illustrated in [143,144,145,146].

## 7. Conclusions

Wireless communication systems can be used in various domains, such as healthcare, finance, transportation, and so on, due to the high data rates and lower latency specifications. The mm-wave and THz bands are promising solutions for 5G and 6G network technologies. Hence, the need for high frequencies in the range of mm-wave and THz bands is increasing exponentially for tackling the problems of increased traffic demands and network capacity. One of the promising methods for overcoming these drawbacks is the implementation of AI methods for developing new communication protocols and designing appropriate circuits for this range of frequencies. In this work, after explaining the definition of AI, various reported studies in the range of mm-wave and THz band frequencies in which AI methods are being used are presented. Additionally, a roadmap for improving concepts related to these high-frequency bands through AI methods is introduced by the authors. Any researcher who reads this work will get a general view about the implementation of networks for various designs at mm-wave and THz frequencies and will be able to select the right one more easily.

## Figures and Tables

**Table 1:** Summary of recently published surveys related to AI methods along with optimizations used in mm-wave and THz bandwidths.

Ref	Year	Content Description of Each Survey
[35]	2021	Describing technologies for 6G wireless communications networks with focus on implementing AI methods at mm-wave and THz bandwidths
[36]	2022	Summarizing mm-wave beamforming training schemes through the ML approaches
[37]	2022	Summarizing THz detection, imaging techniques and ML methods
[38]	2022	Explaining ML methods for antenna designs at mm-wave and THz bandwidths
[39]	2023	Describing the AI-based methods used in blockchain approach at 6G and beyond
[40]	2023	Presenting the beamforming techniques based on AI methods for mm-wave communications
[41]	2024	Presenting the ML methods employed for handover optimizations in 5G networks
[42]	2024	Providing blockchain-based method for dynamic spectrum sharing that can be applied at THz bandwidth by ML approaches
[43]	2024	Describing optimizations for multicasting in mm-wave and sub-THz networks
[44]	2024	Summarizing reconfigurable intelligent surface concept for optical systems
[45]	2025	Providing a summary for mm-wave radar-based sensing approaches and applications in autonomous vehicles, smart homes, and industry
This work	2025	(1) Summarizing AI-methods applied in mm-wave and THz bandwidths; (2) introducing types of ML methods for various applications; (3) describing the applications of AI technology at circuit level and/or system level; (4) presenting a future direction for the mm-wave- and THz-based systems with the help of various ML approaches.

**Table 2:** Summary of various types of NNs in recently published works.

Ref.	Target(s)	Type of NN
[52]	Estimating the best beam for 6G networks in the fastest way	DNN
[53]	Improving the network coverage in terms of furthest uplink distances	ML-based physical-layer receiver
[54]	Presenting a mitigation approach for adversarial attacks in 6G networks	Adversarial learning with AI methods
[55]	Predicting the path loss at 28 GHz for 5G networks	ML with GA methods
[56]	Selecting network with subchannel allocation	Deep reinforcement learning
[57]	Presenting energy-efficient method for 6G mobile network	ML-based method
[58]	Estimating beam at mm-wave	ML-based method
[59]	Presenting high-accuracy on-chip calibration method	Thru-reflect-line neural network
[60]	Designing high-gain dipole antenna with its array filter-antenna at mm-wave frequencies	Alternating direction method of multipliers and Bayesian optimization
[61]	Selecting the suitable beam	Long short-term memory (LSTM)-based DNN
[62]	Identifying mm-wave channel states and scenarios	DNN
[63]	Estimating mm-wave channel	CNN
[64]	Generating suitable beamforming configurations with respect to spatial distribution of throughput demand	ML based on the k-nearest neighbors
[65]	Predicting beam-space channel	DNN
[66]	Erasing the behavior privacy in radio frequency signals	DNN
[67]	Providing an effective beam steering functionality with the help of reconfigurable intelligent meta-surfaces	ML with GA
[68]	A method for side link-assisted multiple-mode mmWave scheduling	ML
